# WITHIN-PERSON RELATIONSHIP BETWEEN MINDFULNESS AND COGNITION IS MEDIATED BY SUBJECTIVE AGE

**DOI:** 10.1093/geroni/igac059.345

**Published:** 2022-12-20

**Authors:** Lyndsey Graham, Shevaun Neupert

**Affiliations:** North Carolina State University, Raleigh, North Carolina, United States; North Carolina State University, Raleigh, North Carolina, United States

## Abstract

Cognitive functioning fluctuates daily throughout adulthood. Lapses in mindfulness can have cognitive consequences, which may be impacted by how old a person feels each day. Subjective age was examined as a mediator in the within-person relationship between mindfulness and cognition. 107 younger adults (aged 18-36, M = 19.96) and 116 older adults (aged 60-90, M = 64.71) completed reports of mindfulness and subjective age and tests of inductive reasoning and episodic memory for 8 consecutive days. Within-person multilevel mediation models indicated that daily subjective age mediated the relationship between daily mindfulness lapses and both indicators of daily cognition across ages. However, the mediation effect was stronger for younger adults on inductive reasoning but was stronger for older adults on episodic memory. These results show that daily changes in subjective aging are an important mechanism for daily cognition, with differential impact based on age and cognitive component.

